# Effects of a school-based intervention on active commuting to school and health-related fitness

**DOI:** 10.1186/s12889-016-3934-8

**Published:** 2017-01-05

**Authors:** Emilio Villa-González, Jonatan R. Ruiz, Jason A. Mendoza, Palma Chillón

**Affiliations:** 1 Department of Physical Culture, School of Health Sciences, National University of Chimborazo, Avda. Antonio José de Sucre, Km. 1 1/2 vía a Guano, Riobamba, Ecuador; 2 PROFITH “PROmoting FITness and Health through physical activity” research group, Department of Physical Education and Sport, Faculty of Sport Sciences, University of Granada, Ctra. Alfacar, s/n, 18011, Granada, Spain; 3 Department of Education, Faculty of Education Sciences, University of Almería, Ctra. Sacramento, s/n, 04120. La Cañada, Almería, Spain; 4 Department of Pediatrics, University of Washington School of Medicine, Seattle, USA; 5 Center for Child Health, Behavior and Development. Seattle Children’s Research Institute, Suite 400, M/S: CW8-6, PO Box 5371, Seattle, WA 98145-5005 USA

**Keywords:** Physical activity, Active transport, Public health, Cardiovascular fitness

## Abstract

**Background:**

Active commuting to school has declined over time, and interventions are needed to reverse this trend. The main objective was to investigate the effects of a school-based intervention on active commuting to school and health-related fitness in school-age children of Southern Spain.

**Methods:**

A total of 494 children aged 8 to 11 years were invited to participate in the study. The schools were non-randomly allocated (i.e., school level allocation) into the experimental group (EG) or the control group (CG). The EG received an intervention program for 6 months (a monthly activity) focused on increasing the level of active commuting to school and mainly targeting children’s perceptions and attitudes. Active commuting to school and health-related fitness (i.e., cardiorespiratory fitness, muscular fitness and speed-agility), were measured at baseline and at the end of the intervention. Children with valid data on commuting to school at baseline and follow-up, sex, age and distance from home to school were included in the final analysis (*n* = 251). Data was analyzed through a factorial ANOVA and the Bonferroni post-hoc test.

**Results:**

At follow up, the EG had higher rates of cycling to school than CG for boys only (*p* = 0.04), but not for walking to school for boys or girls. The EG avoided increases in the rates of passive commuting at follow up, which increased in the CG among girls for car (MD = 1.77; SE = 0.714; *p* = 0.010) and bus (MD = 1.77; SE = 0.714; *p* = 0.010) modes. Moreover, we observed significant interactions and main effects between independent variables (study group, sex and assessment time point) on health-related fitness (*p* < 0.05) over the 6-month period between groups, with higher values in the control group (mainly in boys).

**Conclusion:**

A school-based intervention focused on increasing active commuting to school was associated with increases in rates of cycling to school among boys, but not for walking to school or health-related fitness. However, the school-based intervention avoided increases in rates of passive commuting in the experimental group, which were significantly increased in girls of the control group.

## Background

The increasing prevalence of obesity in Spain is a major public health problem. Data from Spain reveals the alarming increase of the prevalence of obesity in children and youth, with a 15.9% of obesity prevalence in children aged from 6 to 9 years [1]. An analysis of the food habits of this population describes that the energy consumption has not increased in the recent past. As such, the decrease in energy expenditure should have association with the increasing prevalence of obesity. To address obesity, the Spanish Ministry of Health and Consumer Affairs drew up the Strategy for Nutrition, Physical Activity and the Prevention of Obesity (NAOS), which aims to promote healthy diets and encourage physical activity by all citizens, with special emphasis on children. Currently, there is evidence that active commuting to school (defined as walking and cycling to and from school) may have important health implications for youth. Previous reviews [2, 3] reported a positive association between active commuting to school and overall physical activity levels. Further, active commuting to school has been positively associated with higher cardiorespiratory fitness in young people [4, 5] and there is some evidence of a relationship with other health-related fitness markers [6]. A 12-week school-based intervention study investigated the association between active commuting to school and physical fitness variables, and showed that 10–13 year old children who cycled to school improved their cardiorespiratory fitness [7]. A cross-sectional study showed that 15–19 years old adolescents who bicycled to school had higher aerobic power, muscle endurance and flexibility than walkers or passive commuters [4].

The Spanish rates of active commuting to school have declined dramatically over the past several years [8], and belonging to a large family was a main correlate of active commuting to school among Spanish children. Initiatives such as Safe Routes to School, the Walking School Bus, the Walk to School, or the School Travel Plan program, have been implemented to increase children’s walking and cycling to school with some success. A systematic review [9] concluded that more research with higher quality study designs and measures should be conducted to determine the most successful strategies for increasing active commuting to school. Some intervention studies reported an increase in the percentage of active commuting to school; however, the degree of change varied widely from 3 to 64% [9].

The main objective of the present study was to investigate the effects of a school-based intervention on active commuting to school and health-related fitness in school-age children of Southern Spain. Our hypothesis was that a school-based intervention focused on increasing the frequency of active commuting to and from school would increase the levels of active commuting to school and thereby improve health-related fitness.

## Methods

### Participants

A total of 494 children aged 8 to 11 years were invited to participate in the study. From these, 469 children (94.9% of the total sample: 251 boys, 218 girls) were included in the final analytic sample due to having valid data on commuting to school. Participants were recruited from five public schools in the provinces of Granada (Salobreña, *n* = 119; Huétor Vega, *n* = 80; Santa Fe, *n* = 96; the city of Granada, *n* = 128) and Jaén (Castillo de Locoubín, *n* = 46) to participate in an intervention to increase walking and cycling to school. Children with valid data on commuting to school at baseline and follow-up, sex, age and distance from home to school were included in the final analysis (*n* = 251; 50.8% of the invited sample). Non-random allocation to experimental group (EG) or control group (CG) was performed at the school-level.

### Study design

**Fig. 1:**
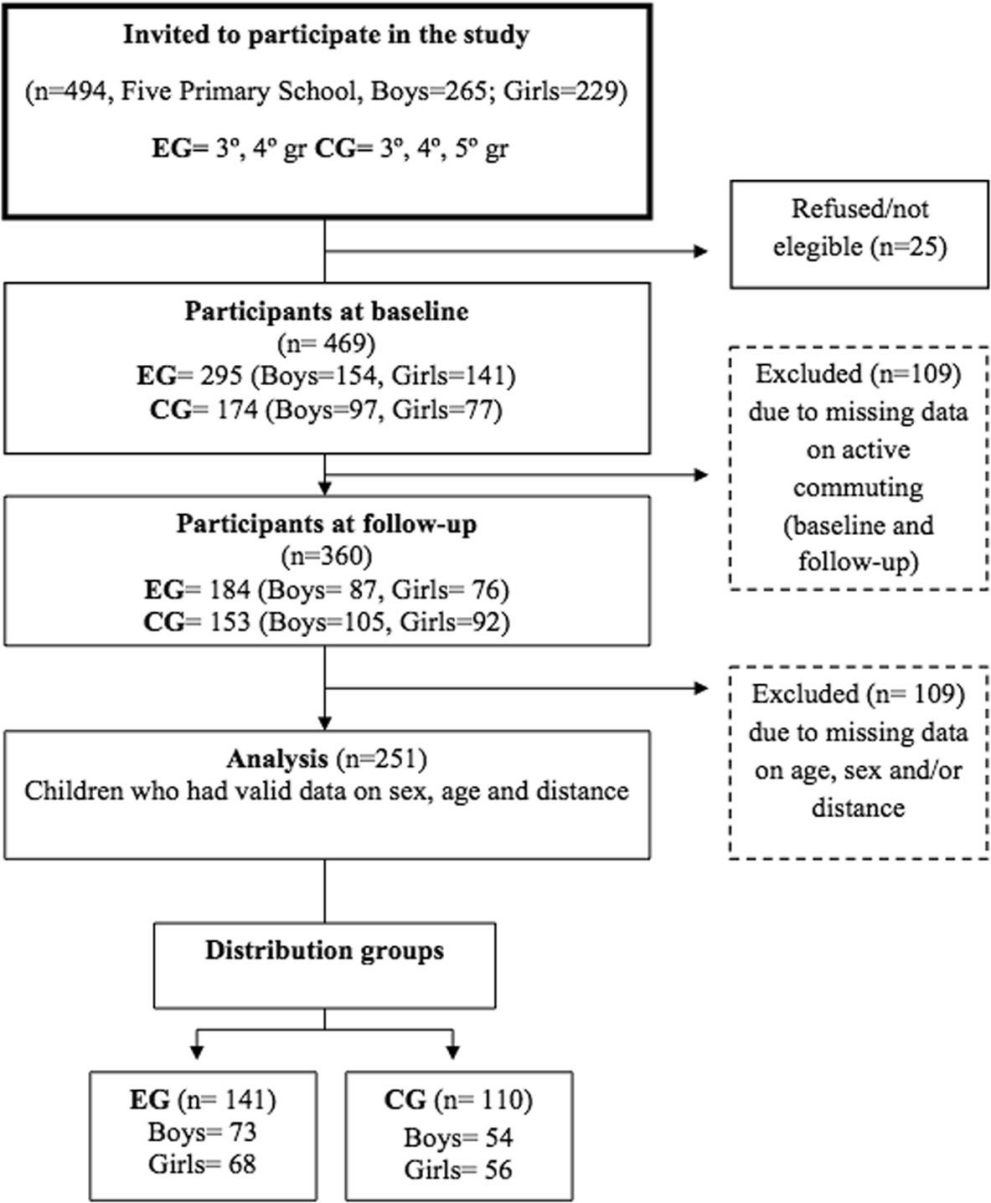
Flow chart corresponding to the participants included at baseline, follow-up and in the analysis. *CG*, control group; *EG*, experimental group

We conducted a quasi-experimental trial with a total of 141 participants from 3 schools in Salobreña, Huétor Vega and Santa Fe, which took part in the experimental group (EG) and received a 6-month intervention program focused on increasing active commuting to school. A total of 110 participants from 2 schools in Granada and Castillo de Locoubín took part in the control group (CG), who did not receive the intervention. EG schools were assigned by the local government (Diputación de Granada) and municipalities. Control schools were selected for comparison by the researchers based on having similar characteristics (socioeconomic level and setting – urban vs. rural) as the experimental schools. Participants did not know the assignment prior to being recruited. All the schools were public and primary education schools within the National Educational System. A flow chart corresponding to the study design is shown in (Fig. 1). The outcome measures were taken during school days prior to (baseline) and after (follow-up) the intervention program in the months of January and June of the academic year 2011/2012 in every school, respectively. All measurements were taken in the same period and the five schools belonged to the same region and had similar weather conditions, i.e., average temperature in January was 10 °C and in June was 20 °C (https://www.wunderground.com). The study was conducted within a public health initiative lead by Diputación de Granada (Área de Medio Ambiente). The purpose of this program was to promote safe and healthy ways of commuting from home to school. An agreement was signed by the school board (decision-making body of a school), Diputación de Granada and the municipalities. The school board, parents and students were informed about the study and they agreed to participate.

### Intervention

**Fig. 2:**
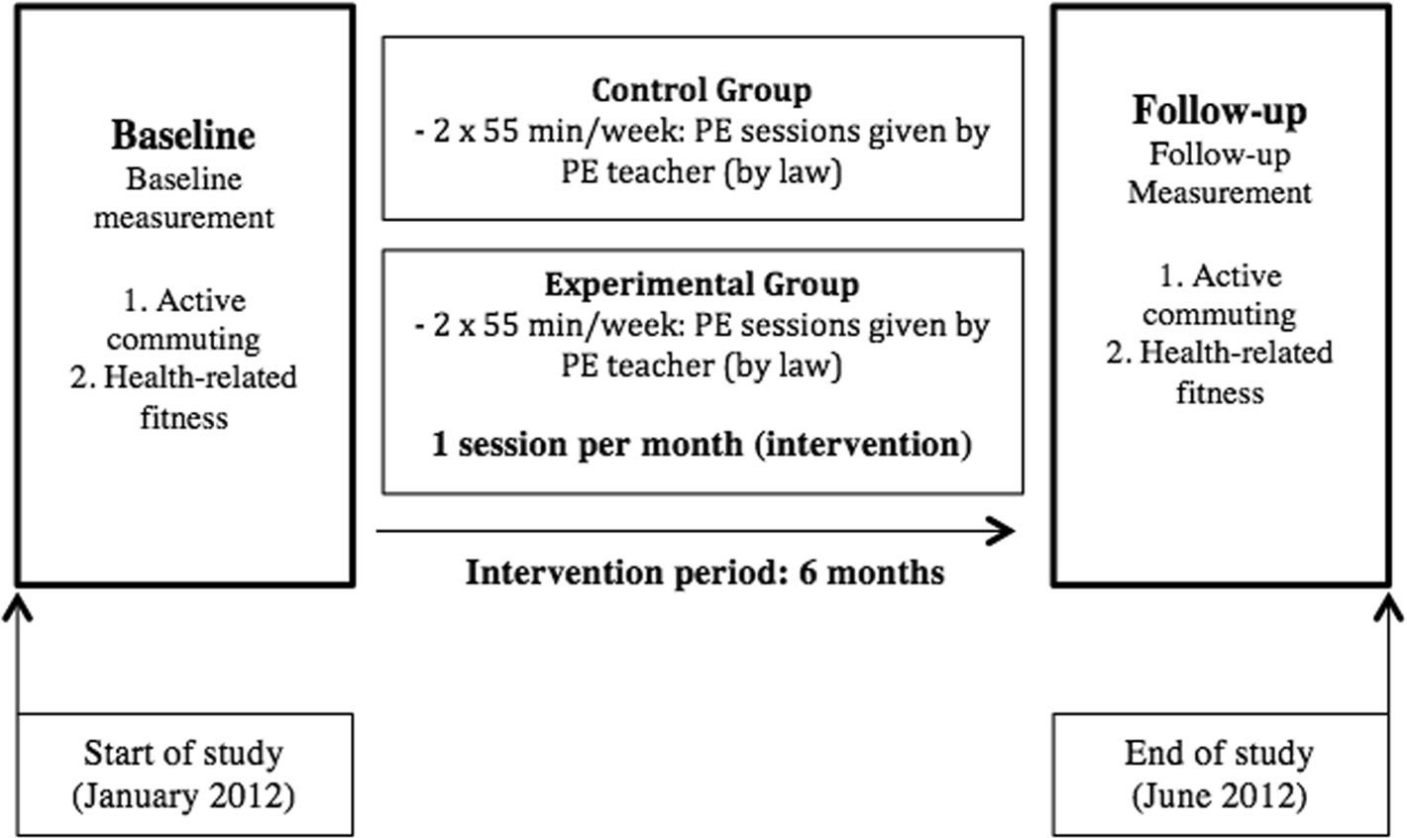
Summary of the study design. *PE*, Physical Education

The intervention focused on increasing the frequency of active commuting to and from school among children. Both teachers and the research team implemented the intervention program at each school. The intervention period lasted two school terms (6 months) from January to June of 2012. Children from the EG participated in 6 monthly activities within the intervention program (each activity ranged from 60–120 min each month) during school hours in addition to their regular Physical Education lessons. The intervention included: (i) introductory activities such as a questionnaire on the mode of commuting to school reported by families (parents or grandparents). The objective of this activity was to know how the families commuted in his/her youth and consider whether currently, there were barriers to active commuting to school for their children. (ii) Reading a story and performing scenes related to active commuting to school. The objective of this activity was to familiarize the children with active commuting to school and the neighborhood. (iii) Activity on knowledge about the environmental characteristics around the school. The objective was to know the urban environment (measuring the size of sidewalks and crosswalks, understanding the traffic signs) in the area surrounding the school. (iv) Activity on road safety. The objective was to promote road safety, and analyze the relation between vehicles (cars and bikes) and pedestrians (supporting older people to cross crosswalks, interviewing pedestrian and drivers, warnings for inadequate behaviors of motor vehicle drivers and cyclists) (v) Activity on behaviors in the street. The objective was to know the appropriate behaviors of pedestrians, vehicles and traffic police (measuring the time to cross a crosswalk and the vehicle’s speed, collaborating with the traffic police) (vi) Activity on traditional games. The objective was to practice traditional games that were adapted to the topic of road safety education and active commuting to school (playing cooperative games integrating ethical and social behaviors as if they were citizens and traffic police). A summarized scheme of the study design is presented in (Fig. 2). These activities were carried out in the classroom (i to ii) and in the school neighborhood (iii to vi). Children in the CG and EG received the usual Physical Education sessions according to the National Education Program in Spain, i.e. 55 min sessions twice per week.

### Mode of commuting to school

Participants completed a self-report questionnaire regarding the latest weekly patterns of commuting to and from school (Monday to Friday). The questionnaire has been proposed as the most appropriate measurement for asking about mode of commuting to school after reviewing 158 studies within the scientific literature [10]. The questionnaire included sociodemographic data and a question about the frequency and mode of commuting: *How did you commute to and from school the last week?.* The modes of commuting were: 1) walking, 2) cycling, 3) car, 4) motorcycle or 4) bus. While this particular question for assessing commuting to school has not been formally validated, it is very similar to other 1-item questionnaires on children’s commuting to school that have demonstrated acceptable validity in this age group [11–13]. Walking and cycling were categorized as active commuting, whereas travelling by car, motorcycle and bus were categorized as passive commuting. Children completed the questionnaire with the help of the teacher and the research team in about 20 min. The weekly frequency of commuting to school was expressed as numbers of active travels per week to and from school (range: 0 to 10). A Spanish and English version of the questionnaire is provided at http://profith.ugr.es/pages/investigacion/recursos/paco.

### Health-related fitness

Physical fitness was assessed by the ALPHA health-related fitness test battery of high priority [14], which is a valid and reliable fitness test battery for children and adolescents. All participants completed the fitness tests during physical education class. The same researchers performed all the measurements. Measurements were organized in a circuit, and participants performed each test consecutively, except the cardiorespiratory fitness test where several participants performed it at the same time.

Cardiorespiratory fitness was assessed by the *20-m shuttle run test* [15]. In brief, the child was required to run between 2 lines 20 m apart while keeping pace with audio signals emitted from a prerecorded CD. The initial speed was 8.5 km/h, and the speed was increased by 0.5 km/h per minute. The test was completed when the participants failed to reach the end lines concurrent with the audio signals on 2 consecutive occasions, or when the participants stopped because of fatigue. The equations of Leger [15] previously validated in children and adolescents, were used to estimate maximum oxygen consumption (VO_2max_) from the test scores.

Lower body muscular fitness was assessed by means of the *standing long jump*. The child stood behind the starting line, with feet together, and pushed off vigorously and jumped forward as far as possible. The distance was measured from the takeoff line to the point where the back of the heel nearest to the takeoff line landed on the mat or non-slippery floor. The test was repeated twice, and the best score was retained (in cm).

Upper body muscle strength was assessed by means of *handgrip strength* using a hand dynamometer with adjustable grip (TKK 5401 Grip D; Takey, Tokyo, Japan). Children were given a brief demonstration and verbal instructions for the test and, if necessary, the dynamometer was adjusted according to the child’s hand size. The test was done in the standing position with the wrist in the neutral position and the elbow extended. Children were given verbal encouragement to ‘squeeze as hard as possible’ and apply maximal effort for at least 2 s (sec). Two attempts per hand were performed. The average of the best scores achieved by each hand was used in the analysis.

Speed-agility was measured by the *4×10 shuttle run test*. Two lines, at a distance of 10 m, and two cones were placed at the distant line. The participants ran as fast as possible from the starting line, picked up one sponge at the distant line, returned to the start line and placed the cone on this line, before repeating the same run and retrieving the second sponge. Two attempts were performed, and the best score was retained (in sec). The agility measure was the time to complete this 40-m run with correct placement of the cones.

### Distance to school

*The distance from home to school* was estimated using the Internet program Google Maps V.6 [16]. The shortest network path between each student’s home address and school was measured in meters.

### Statistical analyses

Differences in socio-demographics characteristics (sex, age and distance from home to school) between included and excluded participants were compared using the chi-square test for categorical variables and Mann–Whitney *U* test for non-normal continuous variables. Differences in the distance from home to school between CG and EG were tested using the Mann–Whitney *U* test. The normality of the fitness test variables was tested using the Kolmogorov-Smirnov test. The participants were analyzed by study group (CG or EG), sex (boys and girls) and assessment time point (baseline and follow-up). Thus, a 2×2×2 Factorial ANOVA was used to analyze the interactions and main effects of the three independent variables or factors (study group, sex and assessment time point) on frequency of active commuting, modes of commuting and health-related fitness. Age and distance were used as covariates. Post-hoc comparisons were performed by Bonferroni test. Level of significance was set at 0.05. Analyses were performed using the PASW (v. 20.0 for Windows, Chicago, IL, USA).

## Results

### Socio-demographics characteristics

There were significant differences between included and excluded participants for age only; included participants had a lower age than excluded participants (9.13 vs. 9.51 years; *p* < 0.01). No other differences between included and excluded participants were observed for sex and distance to school. Examining baseline data, there were differences between the CG and EG for distance from home to school whereby the CG had a greater distance than the EG (1467.0 vs. 604.6 m; *p* < 0.01). Children with valid data on commuting to school at baseline and follow-up, sex, age and distance from home to school were included in the final analysis (*n* = 251).

### Interactions and main effects of study groups, sex and assessment time point on frequency of active commuting and on modes of commuting

No significant interactions between any independent variable (study groups, sex and assessment time point) on frequency of active commuting (number of active travels per week) and on modes of commuting (number of travels per week) were observed. However, a significant main effect of the independent variable study groups was detected on the frequency of active commuting (F[1, 565] = 15.04, *p* < 0.001, ƞ^2^ *p* = 0.02), and several modes of commuting: walk (F[1, 565] = 14.41, *p* < 0.001, ƞ^2^ *p* = 0.025), car (F[1,565] = 9.49, *p* = 0.002, ƞ^2^ *p* = 0.01) and bus (F[1,565] = 11.08, *p* = 0.001, ƞ^2^ *p* = 0.019). The Bonferroni pair comparisons of active commuting to school and mode of commuting by study group, sex and assessment time point are presented in Table 1.

**Table 1:** Active commuting to school and mode of commuting by study group, sex and assessment time point

	Study groups	Sex	Assessment time point	n	Mean	SD
Active commuting						
Frequency of active commuting (n°/week)^a^	EG	boys	Baseline	114	5.9	0.35^b^
			Follow-up	73	5.7	0.45
		girls	Baseline	114	5.7	0.35^b^
			Follow-up	68	5.5	0.46
	CG	boys	Baseline	49	4.1	0.55
			Follow-up	54	4.7	0.53
		girls	Baseline	47	4.1	0.55
			Follow-up	56	4.2	0.51
Mode of commuting (n°/week)^a^						
Walk	EG	boys	Baseline	114	5.7	0.35^b^
			Follow-up	73	5.4	0.45
		girls	Baseline	114	5.6	0.35^b^
			Follow-up	68	5.4	0.46
	CG	boys	Baseline	49	3.7	0.55
			Follow-up	54	4.6	0.52
		girls	Baseline	47	4.0	0.55
			Follow-up	56	4.2	0.51
Bike	EG	boys	Baseline	114	0.2	0.07
			Follow-up	73	0.3	0.09^b^
		girls	Baseline	114	0.1	0.07
			Follow-up	68	0.1	0.09
	CG	boys	Baseline	49	0.3	0.11
			Follow-up	54	0.0	0.11
		girls	Baseline	47	0.1	0.11
			Follow-up	56	0.0	0.10
Car	EG	boys	Baseline	114	3.3	0.36
			Follow-up	73	3.1	0.46
		girls	Baseline	114	3.5	0.36
			Follow-up	68	3.2	0.47
	CG	boys	Baseline	49	4.4	0.56
			Follow-up	54	3.9	0.54
		girls	Baseline	47	4.5	0.57
			Follow-up	56	5.0	0.52^b^
Motorcycle	EG	boys	Baseline	114	0.2	0.07
			Follow-up	73	0.1	0.08
		girls	Baseline	114	0.1	0.06
			Follow-up	68	0.1	0.08
	CG	boys	Baseline	49	0.1	0.10
			Follow-up	54	0.2	0.10^c^
		girls	Baseline	47	0.1	0.10
			Follow-up	56	0.0	0.09
Bus	EG	boys	Baseline	114	0.2	0.17
			Follow-up	73	0.4	0.22
		girls	Baseline	114	0.2	0.17
			Follow-up	68	0.2	0.22
	CG	boys	Baseline	49	0.7	0.26
			Follow-up	54	0.9	0.25
		girls	Baseline	47	0.7	0.26
			Follow-up	56	1.1	0.24^b^

Data are shown as mean and standard deviation adjusted by age and distance

*EG* experimental group, *CG* control group, *SD* standard deviation

^a^Number of active travels to and from school per week (Range: 0–10). Factorial ANOVA analysis (study group, sex and assessment time point). Pair comparisons by Bonferroni test. Statistical differences (*p* < 0.05). Statistical signals are presented in the higher mean of the pair comparisons: ^b^EG vs CG; ^c^boys vs girls

### Interactions and main effects of study groups, sex and assessment time point on health-related fitness

Several significant interactions in all health-related fitness tests were observed. Significant interactions between study groups and sex, and between study groups and assessment time point were observed for VO_2max_ (F[1,486] = 18.92, *p* < 0.001, ƞ^2^ *p* = 0.03 and F[1, 486] = 13.44, *p* < 0.001, ƞ^2^ *p* = 0.02, respectively), 20-m shuttle run test (F[1, 489] = 19.81, *p* < 0.001, ƞ^2^ *p* = 0.03 and F[1, 489] = 12.33, *p* < 0.001, ƞ^2^ *p* = 0.02, respectively), and standing long jump test (F[1, 490] = 6.74, *p* = 0.010, ƞ^2^ *p* = 0.01 and F[1, 490] = 7.11, *p* = 0.008, ƞ^2^ *p* = 0.01, respectively). Significant interactions between study groups and assessment time point was observed for handgrip strength test (F[1, 495] = 14.48, *p* < 0.001, ƞ^2^ *p* = 0.03). In the 4 × 10 shuttle run test a significant interaction between study groups and sex was observed (F[1, 489] = 6.41, *p* = 0.01, ƞ^2^ *p* = 0.01).

Main effects of the study groups and sex were observed in VO_2max_ (F[1,489] = 6.92, *p* = 0.009, ƞ^2^ *p* = 0.01 and F[1, 489] = 51.43, *p* < 0.001, ƞ^2^ *p* = 0.09, respectively) and in 20-m shuttle run test (F[1,489] = 7.93, *p* = 0.005, ƞ^2^ *p* = 0.04 and F[1, 489] = 59.97, *p* < 0.001, ƞ^2^ *p* = 0.02, respectively). Main effects of the assessment time point were observed in 20-m shuttle run test (F[1,489] = 12.51, *p* < 0.001, ƞ^2^ *p* = 0.02) and 4 × 10 shuttle run test (F[1,489] = 8.08, *p* = 0.005, ƞ^2^ *p* = 0.02). Main effects of sex were observed for standing long jump test (F[1,490] = 36.39, *p* < 0.001, ƞ^2^ *p* = 0.07), handgrip strength (F[1,495] = 4.12, *p* = 0.043, ƞ^2^ *p* = 0.08), and 4 × 10 shuttle run test (F[1,489] = 24.06, *p* < 0.001, ƞ^2^ *p* = 0.04). The Bonferroni test for health-related fitness by study groups, sex and assessment time point are presented in Table 2.

**Table 2:** Health-related fitness by study groups, sex, assessment time point

Health-related fitness	Study groups	Sex	Assessment time point	n	Mean	SD
Cardiorespiratory Fitness						
VO2max (mL/kg per min)	EG	boys	Baseline	96	42.8	0.41^b, c^
			Follow-up	70	41.2	0.45
		girls	Baseline	85	41.2	0.45
			Follow-up	61	40.5	0.49
	CG	boys	Baseline	47	43.5	0.62^b^
			Follow-up	44	46.0	0.63^a, c^
		girls	Baseline	43	39.6	0.61
			Follow-up	50	40.9	0.59
20-m suttle run (stage)	EG	boys	Baseline	96	2.2	2.22^b^
			Follow-up	70	2.2	2.17
		girls	Baseline	86	1.8	1.76
			Follow-up	61	1.8	1.82
	CG	boys	Baseline	48	2.5	2.54^b^
			Follow-up	45	3.7	3.70^a, b, c^
		girls	Baseline	43	1.3	1.34
			Follow-up	50	1.9	1.91^c^
Muscular Fitness						
Standing long jump (cm)	EG	boys	Baseline	96	120.2	2.03^c^
			Follow-up	70	116.0	2.41^b^
		girls	Baseline	87	115.7	2.12^a^
			Follow-up	62	107.9	2.54
	CG	boys	Baseline	48	117.7	2.93^b^
			Follow-up	44	123.1	3.04^b^
		girls	Baseline	43	103.4	3.04
			Follow-up	50	105.6	2.81
Hand-grip strength (kg)	EG	boys	Baseline	97	14.5	0.48
			Follow-up	70	13.9	0.57
		girls	Baseline	89	14.4	0.50
			Follow-up	63	13.3	0.60
	CG	boys	Baseline	48	13.7	0.70
			Follow-up	45	16.9	0.72^a, b, c^
		girls	Baseline	43	12.9	0.72
			Follow-up	50	14.7	0.67
Speed-agility						
4×10 shuttle run (sec)	EG	boys	Baseline	95	13.7	0.14
			Follow-up	69	13.6	0.17
		girls	Baseline	87	14.0	0.14
			Follow-up	61	13.9	0.17
	CG	boys	Baseline	47	13.8	0.20^b, c^
			Follow-up	45	13.2	0.20^b^
		girls	Baseline	43	14.8	0.21^a, c^
			Follow-up	49	14.2	0.19

Data are shown as mean and standard deviation adjusted by age and distance

*EG*, experimental group; *CG*, control group; *SD*, standard deviation

Factorial ANOVA analysis (study group, sex and assessment time point). Pair comparisons by Bonferroni test. Statistical differences (*p* < 0.05). Statistical signals are presented in the higher mean of the pair comparisons: ^a^EG vs CG; ^b^ boys vs girls and ^c^baseline vs follow-up

The analyses were repeated using the log-transformation of the frequency of active travels per week (n°/week) and the results remained consistent. The analyses were repeated including school as a covariate and the results remained consistent. Intention-to-treat analysis was used by last observation carry-forward and the results remained constant (data not shown).

## Discussion

The current school-based intervention focused on increasing children’s active commuting to school was associated with a small but significant increase in cycling to school at follow-up only for boys compared to controls (*p* = 0.04), but was not associated with increases in rates of walking to school or health-related fitness for boys or girls. However, the school-based intervention avoided increases in rates of passive commuting in the EG, which were significantly increased in the CG. The increase in passive commuting in the CG was in girls, for car and bus modes. Moreover, we observed significant interactions and main effects between independent variables (study groups, sex and assessment time points) on health-related fitness over the 6-months period between CG and EG, with higher values in the CG, and mainly in boys.

Previous school-based intervention studies that promoted active commuting to school among children have found increases in the rates of active commuting to school (increasing walking or cycling to school) [17, 18], daily physical activity levels (total daily steps) [19–21], and several behaviors (children’s pedestrian safety behaviors) [22, 23]. However, there is little evidence on fitness-related outcomes [7]. Comparisons between studies should be interpreted cautiously, because the scope and content of the interventions to increase active commuting to school, the measurement of the main outcome (active commuting to school) and the socioeconomic and geographical contexts differ [9].

### Active commuting to school

Most of the identified studies that implemented school-based interventions promoting active commuting to school reported a positive effect on the rate of active commuting to school [17–19, 24–28]. The increase in active commuting across these studies ranged from 2 to 63%. Consistent with those studies, our study showed a modest increase in cycling to school at follow-up for boys in the EG. Other studies reported no effect on changing rates of active commuting to school [7, 23, 29], which is consistent with our results, where after intervention, the EG did not increase the frequency of active commuting (walking and cycling together), however, the intervention avoided increases the rates of passive commuting, whereas CG increased passive commuting (car and bus modes) mainly in girls. The lack of change in walking to school could be attributed to the intervention being of insufficient intensity and duration, i.e. one activity per month during 6-months across the intervention such as in the present study may be insufficient to produce behavior change. In Norwegian children, the intervention had no significant impact on cycling to school, since a high rate of children reported cycling to school prior to the intervention [7]. In contrast the present study, which had low rates of cycling to school at baseline, the intervention was associated with a modest but significant increase in cycling to school among boys only *(p = 0.04)*. In Canadian children there was no significant increase for active commuting to school at follow-up after a one-year intervention. However, there was considerable variation in active commuting at the school level [29]. In British children, there was no evidence for changing the mode of commuting at follow-up [23], also possibly due to the low intensity of the intervention (16 h of expert assistance over one school year). Experimental trials that showed significant changes to active commuting to school had more intensive and durable intervention time with the children, e.g. adult-chaperoned walk to school groups offered daily or twice daily [21, 25], rather than once monthly.

### Health-related fitness

In the current study we found a significant difference between groups on physical fitness variables (i.e., cardiorespiratory fitness, muscular fitness and speed-agility) after the intervention with higher values in the CG. To the best of our knowledge there is little evidence of the effect of school-based interventions to increase active commuting to school on health-related fitness. A previous study investigated the effect of a 12-week cycling-to-school intervention on cardiorespiratory fitness, and children who cycled to school improved their cardiorespiratory fitness [7]. At follow up, a significant difference between those starting cycling and those who did not start cycling was observed for VO_2_peak. Different results were observed in the current study, since we found that the EG had significantly greater decreases in VO_2max_ and 20-m shuttle run tests compared to the CG. There is previous cross-sectional evidence that commuting by cycling is associated with expected physical fitness variables such as aerobic power or muscle endurance, and even other unexpected physical fitness variables such as flexibility; however, other physical fitness variables such as muscle strength or speed-agility did not differ between bikers and walkers or passive commuters [4]. In the previous two studies, a high percentage of the participants used cycling for commuting to school and the health-related fitness effects could be studied. In the current study, only a 0.1% of the participants cycled to school, which is too small in size to consider its effects. Most of the participants in the current study walked to school and the walking rate did not show significant differences after the intervention in either the EG and CG. On the other hand, there is previous cross-sectional evidence that walking as a mode of commuting may be insufficient to modify health-related fitness [4, 30]. However, the results in the current study showed that the health-related fitness levels in the CG improved more than in the EG at follow-up. Mostly, boys in both groups (CG and EG) presented better health-related fitness than girls at baseline and follow-up. We speculate that other variables not included in the current study such as differences in total daily physical activity, sexual maturation or dietary intake might explain the observed health-related fitness improvement in the participants from the CG. Furthermore, the CG had a longer distance from home to school than EG; consequently, the CG had to cover longer walking and cycling distances which provide a greater opportunity for improvement in their physical fitness levels compared to the EG. Moreover, since the present study had nonrandom allocation to EG or CG and had substantial participants dropped from analyses, the results may reflect inherent group differences between EG and CG and may not necessarily be entirely attributable to the active commuting to school intervention.

### Intervention analysis

The present intervention was focused mainly on children and not on parents. The intervention was based on the conceptual framework of active travel in children proposed by Panter [31] targeting mainly individual factors such as children’s perceptions (safety perception on the way to school) and attitudes (independence or motivation to walk). The intervention weakly targeted other determinants previously described, such as the urban form or parental perceptions [32–34]. However, Spain is a novel country regarding the implementation and evaluation of interventions to promote active commuting to school and the effectiveness of these programs are still unknown. The inclusion of strategies which promote ACS should be carried out and evaluated progressively. The first step is to conduct interventions focused on changing children’s and families’ perceptions about the awareness of active commuting to school. The second step is to create more ambitious interventions which include changes to the environment and/or provides opportunities to walk to school (e.g. Safe Routes to School encouragement programs). The effectiveness of interventions on active commuting to school is related to three main elements: schools, parents, and communities [9]. These were included in the current school-based intervention study, but the involvement of both parents and communities was weak, and the involvement of the school might be insufficient for success. However, our study showed a modest increase in cycling to school at follow-up for boys in the EG, although we only carry out an activity related to cycling to school (iv: Activity on road safety).

Parents participated in one activity (activity i) and the community participated in two activities (activity iv and v) with support from police, neighbors and municipalities. The interventions with the highest effectiveness [28] reported strong involvement of schools through principals and teachers working actively in the intervention. However, in the current intervention, all schools agreed to participate in the intervention through the principals, but the involvement of some teachers was poor and this could affect the motivation of children to actively commute to school. Furthermore, the dose of the intervention could be insufficient, since this only included one activity per month. Previous successful active commuting to school interventions have provided daily opportunities for the intervention [21, 25]. Another reason for explaining the lack of effectiveness might be the lack of financial support. Several studies had financial support for providing resources and staff for the intervention but in the current study, there was no similar financial support [27].

### Limitations

The present intervention study has limitations and strengths. We conducted a quasi-experimental design (no group randomized allocation) with a self-reported questionnaire administered to children that does not have established validity evidence. There was a relatively small final sample size, with 50% of participants which were dropped from analyses (CG = 63%, EG = 47%) due to missing data mainly on distance from home to school, since children did not provide a full and accurate home address. External validity is limited due to the relatively narrow age range and the small number of schools in cities from a single country, although this information is very important to implement future interventions in Spain. Additionally, there was no information on participant’s socio-economic status (SES) and weather conditions. The method used to estimate home-school distance (Google Maps) may not represent the actual route taken as well as the shortest distance from home to school. Unfortunately, we were not able to measure total daily physical activity or dietary intake, which may influence our results. A major strength of the study is the measurement of different components of fitness through several physical fitness tests and the rigorous analyses of the school-based intervention including both EG and CG that allow comparisons between groups. Future research should focus on how to assist children and their families to use active travel behaviors in the longer term, with a focus on more intensive interventions.

## Conclusions

A school-based intervention focused on increasing active commuting to school was associated with increasing rates of cycling to school among boys only, but was not effective on increasing the rates of walking to school and the health-related fitness. However, the school-based intervention avoided increases to the rates of passive commuting in the experimental group, which were significantly increased in girls of the control group. These findings have important implications for research reporting the implementation of an intervention to promote active commuting to school. Future work should seek to build on this foundation by incorporating more rigorous research designs, including objective measures, random experimental group assignment, and longitudinal sampling.

## Abbreviations

ALPHA: Assessing levels of physical activity
BMI: Body max index
CG: Control group
Cm: Centimeters
EG: Experimental group
Km/h: Kilometers per hour
M: Meters
M^2^: Meters squared
S: Seconds
SD: Standard deviation
SES: Socio-economic status
VO_2max_: Maximal oxygen consumption
WC: Waist circumference
